# Cognitive Functioning and Its Stability Among Young Adults with Perinatal HIV Exposure or Acquisition in a U.S.-Based Longitudinal Cohort Study

**DOI:** 10.1007/s10461-026-05133-2

**Published:** 2026-06-02

**Authors:** Sharon Nichols, Yanling Huo, Patricia A. Garvie, Sara Caniglia, Renee Smith, Kathleen Malee, Megan Song McHenry, Mary E. Paul, Anna-Barbara Moscicki, Guadalupe Morales Avendano, Katherine Tassiopoulos, Megan L. Wilkins, Claire A. Berman, Paige L. Williams

**Affiliations:** 1 Department of Neurosciences, University of California, 9500 Gilman Drive, Mail Code 0935 La Jolla, San Diego, CA 92093 USA; 2 Center for Biostatistics in AIDS Research, Harvard TH Chan School of Public Health, Boston, MA USA; 3 Independent Consultant, Fort Lauderdale, FL USA; 4 Department of Pediatrics, University of Illinois at Chicago, Chicago, IL USA; 5 Department of Psychiatry, Northwestern University, Chicago, IL USA; 6 Department of Pediatrics, Indiana University School of Medicine, Indianapolis, IN USA; 7 Department of Pediatrics, Baylor College of Medicine, Houston, TX USA; 8 Department of Pediatrics, University of California, Los Angeles, Los Angeles, CA USA; 9 Department of Pediatrics, Keck School of Medicine, University of Southern California, Los Angeles, CA USA; 10 Department of Infectious Diseases, St. Jude Children’s Research Hospital, Memphis, TN USA; 11 Department of Epidemiology, Harvard TH Chan School of Public Health, Boston, MA USA; 12 Department of Biostatistics, Harvard TH Chan School of Public Health, Boston, MA USA

**Keywords:** Human immunodeficiency virus (HIV), Perinatal, Cognitive, Adolescents/young adults, NIH toolbox

## Abstract

The growing population of young adults with perinatal HIV acquisition (YAPHIV) or exposure without acquisition (YAPHEU) warrants investigation of ongoing cognitive effects of PHIV and lasting neurodevelopmental consequences of perinatal exposure to HIV and antiretroviral therapy (ART). Within the US-based AMP Up longitudinal cohort study, 608 YAPHIV and 131 YAPHEU completed the NIH Toolbox (NIHT) assessment of cognitive functioning and provided information on mental health, demographic variables, social determinants of health (SDOH), HIV-related health and ARTs. YAPHIV were categorized at entry as YAPHIV-C (history of CDC Class C AIDS-defining criteria or nadir CD4%<15) or YAPHIV-non-C. General linear regression models were fit to examine associations of HIV status with NIHT age-adjusted Fluid, Crystallized and Total Cognition Composite standard scores and change in scores from entry to the three-year follow-up visit. NIHT performance between YAPHEU and all YAPHIV was comparable, with borderline/low average Fluid and average Crystallized Composite scores; however, we observed overall differences among the three groups, with lowest mean scores among YAPHIV-C and highest among YAPHIV-non-C. In adjusted analyses, mean Fluid Composite scores increased longitudinally across groups, with significantly less increase in both YAPHIV groups versus YAPHEU. Differences were not mediated by mental health variables. Few associations with current or change in HIV disease severity were observed. SDOH analyses supported benefits of greater access to social and economic resources. Although results highlight concerns about long-term effects of perinatal HIV among young adults, differences were subtle and emphasize the importance of modifiable factors such as viral control and access to resources.

**Supplementary Information:**

The online version contains supplementary material available at https://doi.org/10.1007/s10461-026-05133-2.

## Introduction

The effect of HIV on brain functioning has been an area of great concern throughout the HIV epidemic. Vigilance remains critically important for those living with perinatal HIV acquisition (PHIV), now aging into adulthood, given that exposure to HIV and antiretroviral therapy (ART) during significant neurodevelopmental periods may compound their effects in a manner not seen with HIV acquisition later in life [1, 2]. As youth age, HIV has the potential to affect cognitive and behavioral development, acquisition of adaptive and academic skills, quality of life, and expected adult independence [1, 3–6].

PHIV was once accompanied by mild to devastating developmental and neurological deficits in infants and children [5]. With the advancement of effective ART, particularly consistent, early access to highly active ART or protease inhibitors (PIs) [7], severe neurodevelopmental effects of PHIV have become uncommon. Yet, recent reviews note continuing concerns about cognitive functioning among youth with PHIV in global functioning and in specific cognitive domains such as executive functioning [1, 5, 8–11]. Enduring “legacy” cognitive impairments noted in these reviews were associated with prior periods of significant immunosuppression, delayed initiation of ART after infancy, and HIV encephalopathy [5, 12]. Even in those virologically controlled with ART, there remains evidence of ongoing adverse effects of HIV-related inflammation and virus-associated neuropathogenesis as well as potential neurotoxicity associated with ART regimens [13, 14]. In addition, lack of availability and/or nonadherence to ART can allow HIV progression and related impacts on neurodevelopment which may vary depending on their timing in the developmental trajectory. These factors emphasize the importance of continued study in this area [8].

As ART now enables youth with PHIV to reach adulthood, it is important to expand research on cognitive functioning to include young adults with PHIV (YAPHIV) and those exposed *in utero* to HIV and ART without acquisition (YAPHEU). Little is known about cognitive functioning among YAPHIV, who differ in key ways from individuals affected by HIV at other life stages (including children and older adults), despite emerging cognitive and neuroimaging studies that suggest important differences in trajectories among youth with and without PHIV [8, 15, 16]. Significant issues that have not been fully addressed and require further longitudinal study include the stability of cognitive functioning in the young adulthood life stage for YAPHIV or YAPHEU and the influence of modifiable factors including HIV disease progression, mental health, substance use, and socioeconomic, social and structural factors.

Understanding the effects of PHIV on cognitive functioning in young adults, and identifying non-HIV-related points of intervention, require determining the most important aspects of PHIV and ART that affect cognition at that developmental life stage. This understanding is also critical for disentangling the effects of PHIV and ART from other powerful influences at multiple levels. For YAPHIV, this means accounting for prenatal exposure to both HIV and, where relevant, ART. Analyses [8-10] have shown that youth with PHIV and PHEU tend to perform similarly on tests of cognitive functioning overall, yet a subset of those with PHIV and prior significant immunosuppression demonstrates lower cognitive functioning related to “legacy effects” noted above. In general, mean performance for those with PHIV, and for PHEU in some studies, tends to fall below that of standardization samples. Understanding this pattern is important to clarify the effects of PHIV and to identify points of prevention and intervention for children with PHIV and PHEU [5, 17].

Beyond perinatal exposure to HIV and its treatment, cognitive development for all children and youth may be influenced by individual and family mental health, substance use, and exposure to abuse, neglect, stress, trauma, and poverty. Individual mental health has the potential to impact all aspects of HIV care and quality of life for those with PHIV [18–21] and is a central feature of neurodevelopmental outcomes in children with PHIV. Results of machine learning analytic approaches suggest that mental health factors are associated with certain cognitive profiles and are a potentially modifiable target for improving cognition in PHIV [22, 23]. Depression, in particular, may affect cognitive functioning either directly, with a key symptom of both depression and HIV-related cognitive impacts being psychomotor slowing, or indirectly by interfering with adherence to ART and medical management [24]. Depression has been noted to affect cognitive functioning among adults with HIV as well as children and adolescents with PHIV [25]. Although depressive symptoms have been associated with differential neurocognitive trajectories among young adults with behaviorally acquired HIV [26], little is known about the potential interrelationships among depression, anxiety and cognition in YAPHIV and YAPHEU, respectively, as these youth age into adulthood and assume greater independence and responsibility for their own health. Similarly, a large body of literature describes the impact of substance use on cognitive functioning in adults with behaviorally acquired HIV across a broad range of substances including alcohol, cannabis, cocaine, opioids and methamphetamine [27–31]. Substance use has been identified as an area of significant concern [32] as it represents a modifiable influence on cognitive functioning. Evidence has shown differential effects of substance use on the developing brain [33–37], and it is an important comorbidity to consider at a time of transition to adulthood with inherent changes in social support structure and access to medical care. Despite this, few published studies address contributions of substance use to cognitive functioning in YAPHIV and YAPHEU as they age into adulthood.

There has been increased recognition that understanding individual health outcomes requires accounting for the social and environmental context within which people live, referred to as social determinants of health (SDOH) [38, 39]. As people affected by HIV are disproportionately exposed to social inequities across economic status, access to medical and mental health care, education, adversity, and trauma, among others, it is particularly important to address these influences on development and quality of life. Although recognition of these factors’ importance in child development and risk for cognitive difficulties later in life has grown, few studies address their influence on young adults affected by PHIV beyond including limited variables, such as race and income, as potential confounders in analyses [40–43]. The influence of SDOH must be considered for a full understanding of the effect of PHIV and HIV exposure on cognition.

For this manuscript, “cognitive functioning” refers to mental processes, including perception, memory, learning, attention, executive functioning, decision making, visual processing, and language that support acquisition and manipulation of knowledge for reasoning, problem solving and other aspects of goal-directed behavior. Cognitive functioning is viewed as dynamic and modifiable with the focus on ways to support and optimize outcomes among young people rather than as a static potential. Its potential impact on health and other outcomes, including tasks critical for adult independence, emphasizes the importance of understanding cognitive functioning and its stability among young adults with PHIV [3, 4, 6, 8]. To this end, the goals of the analyses presented herein were to characterize cognitive functioning over three years among young adults with PHIV or PHEU; to examine the associations of cognitive functioning and cognitive stability with markers of current and historical HIV disease severity, considering mental health and substance use as potential modifiable mediators; and to explore associations with socioeconomic and social determinants of health.

## Methods

### Study Population

Participants were enrolled in the Adolescent Master Protocol (AMP) Up study of the Pediatric HIV/AIDS Cohort Study (PHACS) network. AMP Up enrolled young adults with PHIV or perinatal HIV exposure without acquisition (PHEU; *>*18 years old) at 14 clinical research sites in the US, including Puerto Rico. The study protocol included in-person visits at study entry and every three years thereafter, with remote visits annually. AMP Up was approved by the institutional review boards of each clinical site and the Harvard T.H. Chan School of Public Health and complied with the Declaration of Helsinki. Written informed consent was obtained from all participants, or from guardians for those unable to consent for themselves, per respective institutional guidelines.

All YAPHIV and YAPHEU enrolled in AMP Up who had completed the NIH Toolbox^®^ for Assessment of Neurological and Behavioral Function (NIHT) at the entry visit and had at least one valid age-corrected Fluid and/or Crystallized Composite score were included. Those with NIHT assessments completed at year 3 were included in analyses examining changes in NIHT performance from entry to year 3. YAPHIV were further categorized as with (YAPHIV-C) vs. without (YAPHIV-non-C) severe HIV disease progression at entry. Severe HIV disease progression was defined as having a CDC class C designation based on AIDS-defining criteria and/or nadir CD4% < 15% [44, 45]. Data for these analyses were collected between study enrollment in 2014 through October 1, 2023.

### Procedures

Participants completed the examiner-administered NIHT and self-administered questionnaires, described below, at entry and year 3 during in-person visits. They completed an annual web-based survey of demographics, mental health (e.g., depressive and anxiety symptoms), substance use, and SDOH. Viral load, CD4 measures and other health information were abstracted annually from medical records.

### Neurocognitive Functioning Measures

The NIHT is a set of validated, psychometrically sound measures [46], with good test-retest reliability in adults (0.92, 0.86, and 0.90 for Crystallized, Fluid, and Total Cognition, respectively [46]; Fluid Cognition: 0.66–0.85 [47]). It was designed to assess various aspects of neurocognitive functioning [46, 48–51] in two broad domains, Fluid and Crystallized cognition [52, 53]. Fluid cognition represents the capacity for new learning and information processing in novel situations and includes NIHT subtests measuring executive function, attention, episodic memory, working memory, processing speed and cognitive flexibility. Crystallized cognition is more dependent on past learning and experience, including formal schooling, and is evaluated in the NIHT using vocabulary and word reading subtests [49]. The NIHT has been used globally in numerous contexts and has demonstrated sensitivity in distinguishing cognitive differences in populations at risk for poorer outcomes, including those negatively affected by social drivers of health [42, 54]. Primary analyses used age-corrected standard scores for Fluid Cognition Composite, Crystallized Cognition Composite, and Total Composite (including both Fluid and Crystallized components) as outcomes. Secondary analyses used age-corrected standard scores for the NIHT cognition subtests: Flanker, List Sorting Working Memory, Dimensional Change Card Sorting, Pattern Comparison, and Picture Sequence (forming the Fluid Cognition composite) and Picture Vocabulary and Oral Reading Recognition (forming the Crystallized Cognition composite).

### Mental Health and Substance Use

Questionnaire measures of depressive symptoms (Patient Health Questionnaire (PHQ-9 [55] or Center for Epidemiologic Studies Depression Scale (CES-D-10) [56], depending on year of enrollment) and anxiety (Generalized Anxiety Disorder, GAD-7) [57] were interviewer-administered at entry and year 3. Substance use was assessed at both time points via an online survey and included self-reported frequency of use within the past year for alcohol and 30 days for tobacco and marijuana. For substance use and mental health assessments, measures obtained at entry were used in analyses.

### Demographic Information and Social Determinants of Health

Self-reported demographic variables included age at assessment, sex, race, ethnicity, and education (< high school vs. ≥ high school) at entry. Self-reported SDOH were assessed via the online survey at study entry. These variables included annual household income, employment status (none, full-time, part-time), type of medical insurance (public only, private only, public and private, uninsured, unaware), living situation (experienced homelessness in past 6 months vs. not), and negative life events in the past year. Perceived emotional support, instrumental support, friendship, and self-efficacy were self-reported within the NIHT and reported as T-scores; scores obtained at entry were used.

### HIV-Related Health Information for YAPHIV

HIV status was verified at entry. For YAPHIV, data on most recent CD4 count and percent, viral load, and current antiretroviral regimen were obtained by chart review at each annual visit, and historical nadir CD4 count and percent, CDC class, and past encephalopathy diagnosis were obtained at study entry and updated annually. Percent viral load > 1000 copies/mL over the 3 years prior to entry was calculated using all available viral load measures from that time period. ART regimen was classified as integrase strand inhibitor (INSTI)-based, protease inhibitor (PI)-based or non-nucleoside reverse transcriptase inhibitor (NNRTI)-based vs. other or no ART.

### Statistical Methods

Entry demographic and SDOH characteristics were summarized by HIV status. To examine associations of HIV status with entry NIHT scores, we summarized the mean (SD) and median (IQR) NIHT composite and subtest standard scores by HIV status; we then utilized general linear regression models to evaluate the associations between HIV status (three groups: YAPHEU, YAPHIV-C, YAPHIV-non-C) and each separate NIHT composite score, both unadjusted and adjusted for potential confounders. To examine associations of HIV status with changes in NIHT scores from entry to year 3, we descriptively summarized the changes by HIV status and then fit general linear regression models similar to those described above to estimate the unadjusted and adjusted associations. All multivariable models were adjusted for age at time of NIHT assessment, sex, race/ethnicity, and education at entry.

To evaluate potential mediation effects of mental health and substance use at entry on the associations between HIV status and the changes in NIHT scores, we first examined the association between HIV status and each potential mediator; if an association was observed, mediation analyses (unadjusted and adjusted) were then performed. Mediation analyses were conducted among two subset populations to perform pairwise comparisons, namely, YAPHEU vs. YAPHIV-C and YAPHEU vs. YAPHIV-non-C. The hypothesized causal pathway for the mediation analysis assumed PHIV status started at birth, with the mediators of mental health and substance use measured in young adulthood occurring after the exposure (at least 18 years later), and the key outcomes of interest measured after the mediators (~3 years later). For exploratory analyses of the influence of SDOH, we descriptively summarized the relationships of NIHT composite and subtest scores with the SDOH measures at entry. All analyses were conducted with SAS V9.4 (SAS Institute, Cary, NC), and two-sided p-values < 0.05 were considered statistically significant.

## Results

### Study Population

Participant characteristics are presented in Table 1. A total of 739 participants were included in this analysis, 82% (*n* = 608) of whom were YAPHIV, and of those, 57% (*n* = 348) had history of severe disease progression prior to study entry (YAPHIV-C). Mean age at first NIHT assessment was 18.7 and 22.3 years for YAPHEU and YAPHIV, respectively. Half of YAPHEU and nearly two-thirds of YAPHIV were female; two-thirds of the total sample identified as Black, and 30% identified as Hispanic/Latinx. A lower percentage of YAPHEU than YAPHIV completed high school (57% vs. 71%), and YAPHEU less often were employed (39% vs. 45%). Additional information about household income, access to medical insurance, recent homelessness, life events, and NIHT social support scores are presented in Table 1.

**Table 1:** Entry characteristics of AMP up participants with NIH toolbox assessments by HIV status

Characteristic	Total (*n*=739)	YAPHEU (*n*=131)	YAPHIV (overall) (*n*=608)	YAPHIV by disease severity	
				YAPHIV-C (*n*=348)	YAPHIV-non-C (*n*=260)
Age (years) at assessment, mean (SD)	21.6 (4.1)	18.7 (1.4)	22.3 (4.2)	23.1 (4.2)	21.1 (3.9)
Female sex at birth	441 (60%)	65 (50%)	376 (62%)	219 (63%)	157 (60%)
Race					
Black	487 (66%)	82 (63%)	405 (67%)	231 (66%)	174 (67%)
White/Other/Multi-racial	218 (29%)	47 (36%)	171 (28%)	100 (29%)	71 (27%)
Missing	34 (5%)	2 (2%)	32 (5%)	17 (5%)	15 (6%)
Hispanic or Latino	221 (30%)	45 (34%)	176 (29%)	106 (30%)	70 (27%)
Highest degree ≥ high school	508 (69%)	75 (57%)	433 (71%)	255 (73%)	178 (68%)
Employment status					
Does not work	386 (52%)	78 (60%)	308 (51%)	177 (51%)	131 (50%)
Works part time	186 (25%)	35 (27%)	151 (25%)	86 (25%)	65 (25%)
Works full time	137 (19%)	16 (12%)	121 (20%)	68 (20%)	53 (20%)
Annual household/personal income					
≤ 10 K	257 (35%)	55 (42%)	202 (33%)	113 (32%)	89 (34%)
> 10–20 K	98 (13%)	16 (12%)	82 (13%)	54 (16%)	28 (11%)
> 20 K	117 (16%)	17 (13%)	100 (16%)	49 (14%)	51 (20%)
Unknown/not reported	267 (36%)	43 (33%)	224 (37%)	132 (38%)	92 (36%)
Type of medical insurance					
Public only	499 (68%)	79 (60%)	420 (69%)	259 (74%)	161 (62%)
Private (plus other)	83 (12%)	11 (9%)	82 (14%)	41 (12%)	41 (16%)
Uninsured	14 (2%)	0 (0%)	14 (2%)	5 (1%)	9 (3%)
Unaware	100 (14%)	30 (23%)	70 (12%)	34 (10%)	36 (14%)
Experienced homelessness in past 6 months	51 (7%)	4 (3%)	47 (8%)	28 (8%)	19 (7%)
Life Events, N responding					
Total life events endorsed, (median (IQR))	3 (2, 6)	4 (2, 6)	3 (2, 5)	3 (1, 5)	3 (2, 5)
Total negative events endorsed	2 (1, 4)	2 (1, 4)	2 (1, 4)	2 (1, 4)	2 (1, 4)
Any negative financial/legal event	208 (28%)	35 (27%)	173 (28%)	99 (28%)	74 (28%)
Any negative residential event	204 (28%)	32 (24%)	172 (28%)	97 (28%)	75 (29%)
Any negative health/death event	429 (58%)	74 (56%)	355 (58%)	208 (60%)	147 (57%)
Any negative relationship change event	192 (26%)	31 (24%)	161 (26%)	106 (30%)	55 (21%)
Any negative violence exposure event	149 (20%)	34 (26%)	115 (19%)	72 (21%)	43 (17%)
Any negative education event	165 (22%)	39 (30%)	126 (21%)	76 (22%)	50 (19%)
Social Support T-scores (mean (SD))					
Emotional support	44.5 (12.1)	44.5 (12.0)	44.5 (12.1)	44.2 (12.0)	44.9 (12.3)
Instrumental support	45.8 (10.0)	47.7 (8.7)	45.4 (10.2)	44.9 (10.4)	46.1 (9.9)
Friendship	46.1 (12.2)	47.5 (10.9)	45.8 (12.4)	44.9 (12.3)	47.0 (12.5)
Self-efficacy	45.4 (11.0)	45.9 (11.5)	45.3 (10.9)	44.7 (11.0)	46.1 (10.6)
Potential mediators at entry among those with NIH Toolbox assessment at Year 3	(*N*=423)	(*N*=76)	(*N*=347)	(*N*=207)	(*N*=140)
Depressive symptoms at entry	76 (18%)	11 (14%)	65 (19%)	43 (21%)	22 (16%)
Drank alcohol in past 12 months					
Never or less than monthly	299 (71%)	54 (71%)	245 (71%)	138 (67%)	107 (76%)
2–4 times a month or more	90 (21%)	17 (22%)	73 (21%)	49 (24%)	24 (17%)
Rather not answer	24 (6%)	3 (4%)	21 (6%)	14 (7%)	7 (5%)
Smoked cigarettes in past 30 days					
Never/not in past 30 days/once	351 (83%)	62 (82%)	289 (83%)	169 (82%)	120 (86%)
2 or 3 times or more	52 (12%)	10 (13%)	42 (12%)	28 (14%)	14 (10%)
Rather not answer	11 (3%)	2 (3%)	9 (3%)	5 (2%)	4 (3%)
Marijuana use in past 30 days					
Never/not in past 30 days/once	281 (66%)	50 (66%)	231 (67%)	133 (64%)	98 (70%)
2 or 3 times or more	115 (27%)	21 (28%)	94 (27%)	58 (28%)	36 (26%)
Rather not answer	17 (4%)	3 (4%)	14 (4%)	10 (5%)	4 (3%)

### Associations of HIV Status with Entry NIHT Scores

No difference was observed between all YAPHIV vs. YAPHEU in any NIHT composite score at study entry (Table 2). However, when comparing all three groups defined by HIV status and disease severity (131 YAPHEU, 348 YAPHIV-C, 260 YAPHIV-non-C), an overall difference emerged in mean Fluid Composite (F = 5.36, *p* = 0.005) and Total Composite (F = 3.26, *p* = 0.04) scores, with the highest scores in the YAPHIV-non-C group and the lowest scores among YAPHIV-C group (Table 2). While Crystallized Composite scores followed the same pattern, mean scores were not significantly different. In pairwise comparisons, there also was no difference in mean composite scores between YAPHIV-C or YAPHIV-non-C compared to YAPHEU, in either unadjusted or adjusted analyses (Table 3). Regarding NIHT subtests, in adjusted analyses, compared to YAPHEU, YAPHIV-C had lower mean Pattern Comparison Processing Speed score (−5.9 (CI_95%_: −10.4, −1.4), *p =* 0.01), while YAPHIV-non-C had higher mean Dimensional Change Card Sort score and Picture Vocabulary score (5.0 (CI_95%_: 1.1, 8.9), *p* = 0.01 and 3.6 (CI_95%_: 0.7, 6.5), *p =* 0.02, respectively, Supplemental Table 1).

**Table 2:** Age-corrected NIH toolbox composite scores at entry, and changes between entry and year 3 visit, by HIV status

Timing and NIH Toolbox Composite score	YAPHEU *N*, mean (SD)	YAPHIV (overall) *N*, mean (SD)	YAPHIV by disease severity		t^a^	*P*-value^a^	F^b^	*P*-value^b^
			YAPHIV-C *N*, mean (SD)	YAPHIV non-C *N*, mean (SD)				
Entry	(*n*=131)	(*n*=608)	(*n*=348)	(*n*=260)				
Fluid composite	121, 79.9 (16.3)	589, 80.1 (17.0)	336, 78.1 (16.5)	253, 82.7 (17.4)	0.10	0.92	5.36	0.005
Crystallized composite	131, 95.5 (16.7)	599, 95.3 (17.3)	345, 94.7 (17.8)	254, 96.1 (16.6)	−0.21	0.90	0.46	0.63
Total composite	121, 84.6 (16.2)	580, 85.4 (17.0)	333, 83.9 (16.8)	247, 87.4 (17.0)	0.50	0.62	3.26	0.04
Change from Entry to Year 3	(*n*=76)	(*n*=347)	(*n*=207)	(*n*=140)				
Fluid composite	66, 7.9 (15.6)	330, 4.9 (14.5)	197, 5.0 (14.9)	133, 4.7 (13.8)	−1.55	0.12	1.22	0.30
Crystallized composite	75, −0.7 (11.0)	341, −0.7 (14.1)	206, −2.2 (14.0)	135, 1.7 (13.8)	0.03	0.97	3.51	0.03
Total composite	65, 4.7 (12.8)	324, 2.6 (13.1)	196, 2.0 (13.5)	128, 3.5 (12.5)	−1.18	0.24	1.25	0.29

YAPHIV: young adults with perinatal HIV infection; YAPHEU: young adults with perinatal HIV exposure but uninfected

^a^Comparing 2 groups: YAPHEU vs. all YAPHIV, with two-sample t-test

^b^Comparing 3 groups: YAPHEU vs. YAPHIV with severe disease progression (YAPHIV-C) vs. YAPHIV without severe disease progression (YAPHIV-non-C), with one-way ANOVA test (F-test with 2 df)

**Table 3:** Unadjusted and adjusted associations of HIV status with age-corrected NIH toolbox composite scores at entry, and with changes in scores between entry and year 3 visit

Timing and NIH Toolbox Composite score	Unadjusted			Adjusted^a^	
	Mean (95% CI)	Difference vs. YAPHEU Beta (95% CI)	*p*-value	Difference vs. YAPHEU Beta (95% CI)	*p*-value
Entry Visit					
Fluid composite					
YAPHIV-C	78.1 (76.3, 79.9)	−1.8 (−5.3, 1.7)	0.31	−1.8 (−5.5, 2.0)	0.35
YAPHIV-non-C	82.7 (80.6, 84.7)	2.8 (−0.8, 6.4)	0.13	3.1 (−0.6, 6.7)	0.10
YAPHEU	79.9 (76.9, 82.9)	Ref.		Ref.	
Crystallized composite					
YAPHIV-C	94.7 (92.9, 96.5)	−0.8 (−4.2, 2.7)	0.66	−2.0 (−5.7, 1.8)	0.30
YAPHIV-non-C	96.1 (94.0, 98.2)	0.6 (−3.0, 4.2)	0.75	0.2 (−3.5, 3.9)	0.92
YAPHEU	95.5 (92.6, 98.4)	Ref.		Ref.	
Total composite					
YAPHIV-C	83.9 (82.1, 85.7)	−0.7 (−4.1, 2.8)	0.71	−1.6 (−5.3, 2.1)	0.41
YAPHIV-non-C	87.4 (85.4, 89.5)	2.9 (−0.8, 6.5)	0.12	2.6 (−1.0, 6.3)	0.16
YAPHEU	84.6 (81.6, 87.6)	Ref.		Ref.	
Change from Entry to Year 3					
Fluid composite					
YAPHIV-C	5.0 (3.0, 7.0)	−2.9 (−7.0, 1.1)	0.16	−4.7 (−9.2, −0.2)	0.04
YAPHIV-non-C	4.7 (2.2, 7.1)	−3.3 (−7.6, 1.0)	0.14	−4.8 (−9.3, −0.3)	0.04
YAPHEU	7.9 (4.4, 11.4)	Ref.		Ref.	
Crystallized composite					
YAPHIV-C	−2.2 (−4.1, −0.4)	−1.5 (−5.0, 2.0)	0.41	−0.2 (−4.1, 3.7)	0.91
YAPHIV-non-C	1.7 (−0.5, 4.0)	2.4 (−1.3, 6.2)	0.21	3.2 (−0.7, 7.2)	0.11
YAPHEU	−0.7 (−3.8, 2.3)	Ref.		Ref.	
Total composite					
YAPHIV-C	2.0 (0.1, 3.8)	−2.7 (−6.4, 0.9)	0.15	−2.8 (−6.9, 1.3)	0.18
YAPHIV-non-C	3.5 (1.3, 5.8)	−1.2 (−5.1, 2.7)	0.56	−1.5 (−5.6, 2.6)	0.47
YAPHEU	4.7 (1.5, 7.8)	Ref.		Ref.	

YAPHIV: young adults with perinatal HIV infection; YAPHEU: young adults with perinatal HIV exposure but uninfected

^a^A separate general linear regression model was fit for each composite score, adjusting for age at entry, sex, race/ethnicity, and education

### Associations of HIV Status with Change in Cognitive Functioning

Of the entry sample, 423 (57%; 347 YAPHIV and 76 YAPHEU) had completed the year 3 NIHT assessment at the time of these analyses. Participants who did not complete the year 3 assessment differed significantly from those who did complete the year 3 assessment in age and income, with those missing data on average being older and falling in a higher income category (Supplemental Table 2). Changes in NIHT composite scores from entry to year 3 are summarized in Table 2. All three groups experienced an increase in mean Fluid Composite scores (by 4.7 to 7.9 points) over 3 years; however, for mean Crystallized Composite scores, YAPHIV-non-C experienced a smaller increase, and YAPHIV-C and YAPHEU showed slight decreases (F = 3.51, *p =* 0.03*)*. In adjusted analyses (Table 3), both YAPHIV-C (−4.7 (CI_95%_: −9.2, −0.2), *p* = 0.04) and YAPHIV-non-C (−4.8 (CI_95%_: −9.3, −0.3), *p* = 0.04) had a significantly smaller increase in mean Fluid Composite scores over time than YAPHEU. Among subtest scores, Flanker and Pattern Comparison Processing Speed scores also generally increased over time, but the mean increases in these two scores were smaller for YAPHIV-non-C than for YAPHEU in unadjusted (Flanker: −5.3 (CI_95%_: −10.3, −0.3), *p* = 0.04; Pattern Comparison: −7.2 (CI_95%_: −12.8, −1.6), *p* = 0.01) and adjusted (Flanker: −7.2 (CI_95%_: −12.4, −2.0), *p* = 0.01; Pattern Comparison: −8.2 (CI_95%_: −14.1, −2.4), *p* = 0.01) models (Supplemental Table 3). In crystallized subtest scores, YAPHIV-non-C had a mean increase in the Oral Reading Recognition score while YAPHEU had a slight decrease, with an adjusted difference of 5.6 points higher among YAPHIV-non-C vs. YAPHEU (CI_95%_: 0.2, 11.0, *p* = 0.04; Supplemental Table 3).

### Mediation Effects of Mental Health and Substance Use on Associations between Perinatal HIV Status and Change in Cognitive Functioning

Distributions of potential mediators (mental health and substance use) by HIV status are presented in Table 1, among those with year 3 NIHT assessments. No association was observed between HIV status and any of the potential mediators; however, YAPHEU were slightly less likely to have depressive symptoms compared to the two YAPHIV groups (14% vs. 21% for YAPHIV-C and 16% for YAPHIV-non-C). A mediation analysis was conducted to examine the potential mediating effect of depressive symptoms at entry on associations between HIV status and change in NIHT scores. Adjusted mediation analysis results are presented in Supplemental Table 4 comparing YAPHEU to each of the YAPHIV subgroups (YAPHIV-C and YAPHIV-non-C). No mediating effect of depressive symptoms on differences by HIV status in any composite score was observed.

### Associations of Disease Severity and ART with Entry Cognitive Functioning among YAPHIV

Adjusted associations of each disease severity measure with NIHT scores are presented in Table 4 for Fluid, Crystallized Cognition, and Total Composite scores. In general, associations were stronger for historical than for concurrent measures of HIV disease severity. As shown in Table 4, CDC class C, history of encephalopathy and nadir CD4% < 15% were associated with lower Fluid Composite scores (CDC class C: −5.9 (CI_95%_: −8.9, −2.9), *p* < 0.001; history of encephalopathy: −7.3 (CI_95%_: −12.7, −1.9), *p* = 0.01; nadir CD4% < 15%: −4.8 (CI_95%_: −7.6, −2.0), *p* < 0.001) and Total Composite scores (CDC class C: −5.2 (CI_95%_: −8.2, −2.1, *p* < 0.001; history of encephalopathy: −5.5 (CI_95%_: −10.9, −0.0), *p* = 0.05; nadir CD4% < 15%: −4.5 (CI_95%_: −7.3, −1.7), *p* = 0.001). Adjusted associations of disease severity measures with Fluid Cognition subtest scores are shown in Supplemental Tables 5 and with Crystallized Cognition subtest scores in Supplemental Table 6. With regard to measures of disease severity at entry, NNRTI-based ART regimens (vs. INSTI-based) were associated with lower Picture Sequence Memory score; entry CD4 count of 200 – < 500 (vs. ≥ 500 cells/mm^3^) was associated with lower List Sorting Working Memory scores; and entry viral load ≥ 1000 copies/mL was associated with lower Pattern Comparison score. With regard to historical disease severity measures, CDC class C was associated with lower scores on Dimensional Change Card Sort, Flanker, and Pattern Comparison scores; history of encephalopathy was associated with lower Picture Sequence Memory scores; nadir CD4% < 15% was associated with lower Flanker and Pattern Comparison scores; and < 50% viral load > 1000 copies/mL over the 3 years prior to entry was associated with lower Dimensional Change Card Sort and Pattern Comparison scores. In addition, participants with all viral load measures in past 3 years > 1000 copies/mL had significantly lower Pattern Comparison scores. For Crystallized subtest scores, CDC class C and nadir CD4% < 15% were associated with lower Picture Vocabulary score.

**Table 4:** Adjusted associations of HIV disease severity with age-corrected cognition composite scores at entry, among YAPHIV

HIV Disease measure	Level	Fluid		Crystallized		Total	
		Adj. Difference Beta (95% CI)	*P*-value	Adj. Difference Beta (95% CI)	*P*-value	Adj. Difference Beta (95% CI)	*P*-value
Disease severity measures at study entry							
Type of ART regimen	PI-based	−2.3 (−5.6, 1.1)	0.18	3.3 (−0.1, 6.6)	0.05	1.1 (−2.2, 4.5)	0.51
	NNRTI-based	−2.4 (−6.4, 1.7)	0.25	1.0 (−3.1, 5.0)	0.64	−0.9 (−4.9, 3.1)	0.65
	Other	−0.8 (−6.0, 4.5)	0.77	−1.4 (−6.6, 3.9)	0.61	−1.6 (−6.8, 3.6)	0.55
	INSTI-based	(ref)	–	(ref)	–	(ref)	–
CD4 count	< 200 cells/mm³	−0.8 (−5.1, 3.6)	0.72	−4.3 (−8.6, 0.0)	0.05	−3.5 (−7.8, 0.8)	0.11
	200 – < 500 cells/mm³	−2.1 (−5.3, 1.0)	0.19	−2.1 (−5.3, 1.1)	0.20	−2.9 (−6.1, 0.2)	0.07
	≥ 500 cells/mm³	(ref)	–	(ref)	–	(ref)	–
Viral load	< 50 copies/mL	(ref)	–	(ref)	–	(ref)	–
	50 – < 1000 copies/mL	−0.8 (−4.6, 3.0)	0.69	−3.2 (−7.1, 0.6)	0.10	−2.4 (−6.2, 1.3)	0.21
	≥ 1000 copies/mL	−2.2 (−5.5, 1.1)	0.19	−1.3 (−4.7, 2.0)	0.44	−2.5 (−5.8, 0.8)	0.13
CDC class C		−5.9 (−8.9, −2.9)	< 0.001	−3.0 (−6.1, 0.0)	0.05	−5.2 (−8.2, −2.1)	< 0.001
Encephalopathy diagnosis history		−7.3 (−12.7, −1.9)	0.01	−2.3 (−7.8, 3.2)	0.42	−5.5 (−10.9, −0.0)	0.05
Nadir CD4% < 15%		−4.8 (−7.6, −2.0)	< 0.001	−2.7 (−5.5, 0.1)	0.06	−4.5 (−7.3, −1.7)	0.001
Percent of viral load measures > 1000 copies/mL over the prior 3 years	0%	(ref)	–	(ref)	–	(ref)	–
	< 50%	−3.2 (−6.5, 0.0)	0.05	1.6 (−1.8, 4.9)	0.35	−1.2 (−4.5, 2.1)	0.47
	50 – < 100%	−1.6 (−5.4, 2.2)	0.41	−0.5 (−4.3, 3.3)	0.78	−1.7 (−5.5, 2.1)	0.39
	100%	−5.4 (−11.0, 0.2)	0.06	−1.5 (−7.3, 4.2)	0.60	−4.4 (−10.0, 1.2)	0.12
Age at initiating ART, per year increase		−0.1 (−0.3, 0.1)	0.46	0.0 (−0.2, 0.2)	0.95	−0.0 (−0.3, 0.2)	0.74
Changes in HIV disease severity measures from entry to Year 3							
Change in CD4 count (cells/mm³) from entry to Year-3, per one unit increase		0.0 (−0.0, 0.0)	0.27	−0.0 (−0.0, 0.0)	0.29	−0.0 (−0.0, 0.0)	0.90
Change in viral load from entry to Year-3	Remained suppressed	(ref)	–	(ref)	–	(ref)	–
	Remained unsuppressed	0.8 (−3.4, 5.0)	0.71	1.9 (−2.0, 5.8)	0.34	2.4 (−1.4, 6.2)	0.21
	Became unsuppressed	−0.1 (−5.9, 5.7)	0.97	4.3 (−1.0, 9.7)	0.11	4.0 (−1.4, 9.3)	0.14
	Became suppressed	1.2 (−3.7, 6.1)	0.64	0.5 (−4.1, 5.1)	0.84	2.4 (−2.1, 6.8)	0.30

ART Antiretroviral treatment, CDC Centers for Disease Control and Prevention, INSTI Integrase strand transfer inhibitor, NNRTI Non-nucleoside reverse transcriptase inhibitor

A separate general linear regression model was fit for each composite score, adjusting for age at entry, sex, race/ethnicity, and education

To address concerns regarding incomplete historical HIV disease severity data reported among newly enrolled YAPHIV AMP Up participants, the analyses related to historical disease severity were repeated by restricting to YAPHIV who were enrolled in the preceding PHACS AMP study which collected complete lifetime HIV disease history. Overall, the results were similar to those including all YAPHIV (See Supplemental Tables 7–9); some previously observed associations were attenuated and younger age at initiating ART was associated with higher List Sorting Working Memory score (0.9 (CI_95%_: 0.1, 1.7), *p* = 0.03).

### Associations of Changes in HIV Disease Severity with Changes in Cognitive Functioning

Changes in HIV disease severity measures from entry to year 3 by HIV status among YAPHIV are summarized in Supplemental Table 10. Overall, there was little change in CD4 count and viral load suppression status; many participants remained virally suppressed (45%) or remained unsuppressed (22%) between entry and year 3. The median (IQR) change in CD4 count over the 3 years was 8 (−113, 165) cells/mm^3^. Adjusted analyses for the association of changes in HIV disease severity with Fluid, Crystallized, and Total Composite scores are presented in Table 4. No association was observed between the changes in HIV disease severity measures and any NIHT score.

### Associations of Social Determinants of Health with Cognitive Functioning at Entry

Mean Fluid, Crystallized, and Total Composite scores are summarized in Supplemental Table 11 by each of the SDOH measures. White/other/multi-racial non-Hispanic group (vs. Black non-Hispanic), participants who completed at least a high school education, those with higher income, and who received private only medical insurance (vs. public only) tended to have higher mean composite scores than their counterparts. Emotional support, friendship and self-efficacy were positively correlated with all composite scores. We did not observe an association between each type of negative life event, total negative events or total life events with any NIHT score.

## Discussion

This study represents one of few longitudinal descriptions of cognitive functioning among young adults with perinatally acquired HIV or perinatal exposure to HIV and ART without acquisition. We examined cognitive functioning, mental health, and SDOH among 739 YAPHIV and YAPHEU participants who completed the NIHT cognitive battery at study entry, among whom 423 also completed the three-year follow-up visit. Across all groups, including YAPHIV with and without a history of more severe disease (Class C vs. non-C), NIHT Fluid Cognition Composite scores were in the borderline to low average range while Crystallized Composite scores were in the average range. The lowest scores in both domains were observed among YAPHIV with more severe past disease (i.e., Class C), replicating previous findings of the lasting, significant impact of prior severe HIV disease progression on cognitive functioning in young people with PHIV. While cognitive performance was generally comparable between YAPHEU and all YAPHIV within our study, longitudinal analyses revealed that increases in Fluid Composite scores over time were significantly smaller among both YAPHIV groups compared to YAPHEU. This suggests that PHIV may directly or indirectly blunt developmental gains in fluid cognition during the transition to adulthood, highlighting the need for targeted support to optimize long-term cognitive outcomes in this vulnerable population.

Generally, the few studies of cognitive functioning among YAPHIV to date have been cross-sectional or involved small samples, precluding a full consideration of confounding exposures. These have largely supported earlier studies of children and adolescents that found enduring effects of prior HIV disease progression occurring during infancy or childhood (see [1, 8] for review; [5, 12, 58, 59]). Our results continue to support this pattern using a larger sample of an older, seldom studied, young adult age group, with the lowest scores on the NIHT Fluid Cognition Composite observed among the YAPHIV-C group. Interestingly, the highest scores occurred among YAPHIV *without* prior disease progression, with intermediate performance among YAPHEU. Differences, while significant, were relatively subtle, with the highest and lowest composite scores separated by less than one-third of a standard deviation, and thus of uncertain clinical significance. However, their potential downstream effects on functional trajectories may be important and warrant continued study. Although we can only speculate about the reasons for higher performance among YAPHIV-non-C, it should be noted that families of children with PHIV in a high-resource setting such as the U.S. often have access to support services, both clinical and educational, that are not as easily accessible to those with PHEU. For example, pediatric HIV clinics may conduct routine 3-month medical visits with psychosocial support available; such support may have offered young people with PHIV greater experience and training with health literacy and healthcare management, possibly leading to additional practice with executive functions in general. In addition, due to parental loss, children with PHIV who are now reaching adulthood more likely may have been raised in adoptive or surrogate homes with higher income and greater access to resources. This pattern may be different in lower resource settings, as suggested by Dahmani et al. [17] who found larger differences in processing speed between those with and without PHIV in countries with lower gross national income [17]. In our sample, the YAPHIV groups were more likely to have completed high school despite lower cognitive performance among YAPHIV-C, also consistent with greater support.

This study also adds to the extant literature supporting concerns about long-term cognitive changes among those with PHIV (see [8] for review). Longitudinal analyses showed potentially clinically meaningful improvement in fluid cognition scores over time in all groups. However, there was less improvement, relative to the standardization cohort, over time in fluid cognition, particularly processing speed and inhibition, in both groups with PHIV (approximately one-third of a standard deviation) compared to YAPHEU (approximately one-half of a standard deviation). A prior AMP study found no longitudinal differences between groups during adolescence [60]; however, our results with young adults are consistent with Robbins and colleagues’ [15] finding of less improvement in processing speed (i.e., faster responding) over time among YAPHIV than YAPHEU. The causes of this pattern are unknown. Robbins and colleagues suggested that YAPHIV may benefit less from the practice effects that can result from repeated exposure to tests [15, 61]; however, in our study, where assessments are separated by three years, practice effects are likely minimal or absent. Possible mechanisms of the lasting effects of PHIV on the trajectory of functional development, for example, through long-term impacts of early exposure on young adult brain plasticity, persisting white matter injury [58], and/or ongoing immune activation and systemic inflammation contributing to neuroinflammation [13] warrant further study. Notably, current markers of HIV disease severity such as viral load and CD4 count or percent were not associated with cognitive functioning despite previous findings from the PHACS AMP study of associations of verbal learning with current CD4 count [62]; furthermore, HIV disease markers were largely stable over longitudinal follow-up and not associated with change in cognitive performance in the analyses reported here. In general, our longitudinal results are promising in that no group, including YAPHIV-C, showed significant declines in functioning despite consistent viral suppression in only 45% of YAPHIV, with most differences representing less improvement over time. Despite isolated findings of associations between HIV and ART history with individual NIHT subtests (e.g., earlier initiation of ART with better working memory and ART regimen with picture sequencing), no consistent pattern was observed with current or historical ART.

The complexity of neurodevelopment requires consideration of other individual and social influences to more fully understand the role of health risks such as HIV. We considered substance use and mental health as possible mediators of differences from baseline to year 3 between YAPHEU and YAPHIV due to their potential impact on fluid cognition. Substance use at entry did not differ between groups and was not analyzed further. Because those with and without PHIV differed on depressive symptoms at entry, baseline depression symptoms were explored as a possible mediator of differences in change over time and were not found to mediate these differences. Exploration of social determinants of health revealed associations between NIHT performance and indicators of greater access to social and economic resources (non-Black race, completing high school, access to private insurance). Access to “social capital” (higher self-rated emotional support and friendship), as well as self-efficacy, also were associated with higher performance. These findings suggest potentially modifiable SDOH targets to optimize neurodevelopmental outcomes among young adults affected by PHIV.

Separating cognitive functioning into fluid and crystallized components allowed us to move beyond previous studies of PHIV that used global cognitive measures. Our analyses of fluid cognition were consistent with previous studies showing that young people affected by PHIV tend to perform, on average, below standardization samples [5, 17]; here, mean performance was more than one standard deviation below the mean of the standardization sample. Even so, mean scores for all groups were within the average range for crystallized cognition, which is an encouraging finding. It is possible that this may reflect the educational support often provided through individuals’ and/or families’ ongoing engagement with the clinics involved in this study, which may be less impactful for fluid cognition measures.

This study has several advantages that increase confidence in the findings. These include the large size of the cohort enrolled in AMP Up, the majority with very well-characterized medical and social histories. The NIHT is widely used, well standardized and brief. As a tablet-based electronic battery, it is particularly well suited to measuring aspects of fluid cognition that involve speed of processing and is well accepted by young participants. Nonetheless, this study is not without limitations. These include the lack of an HIV-unexposed comparison sample of young adults, increasing the importance of using a standardized measure. Future studies with an unexposed comparison group and expanded consideration of SDOH will further refine our understanding of the role of not only PHIV, but perinatal HIV and ART exposure themselves, in adult outcomes. A further limitation of our study is the lack of caregiver or socioeconomic status information from the home of origin for all participants, an important consideration for future analyses. Other factors known to influence cognition such as sleep, early adversity and trauma, and environmental exposures such as pollution, may be important as adjustment variables for future studies. Generalizability to lower-resource settings is unknown and warrants further study. Loss to follow-up, which may be more likely in young adult participants who are unable to attend visits because they have work/school obligations, have limited transportation, or have other obstacles to engagement, may also limit generalizability and influence findings. During our study, restrictions related to the COVID-19 pandemic prevented research study visits at all U.S. sites for almost a year, contributing to the smaller numbers with NIHT assessments at 3 years; however, these restrictions impacted all participants equally during that year. Last of all, our analysis of change in NIHT scores between entry and 3 years later did not adjust for baseline scores, which can sometimes result in bias if there is regression to the mean; however Glymour et al. [63] show that falsely inflated estimates of exposure effects on changes in cognitive functioning can arise when analyses adjust for baseline cognitive function.

In summary, our findings show that while young adults with HIV exposure and acquisition have similar cognitive functioning to those with exposure alone, in general, those with significant previous HIV disease progression have lower performance on average. However, differences are subtle and of uncertain clinical significance in this age group. Surprisingly, those with well-controlled PHIV had the highest performance, suggesting an encouraging and considerable resilience. Nevertheless, YAPHIV generally showed less improvement in fluid cognition over time than YAPHEU, a finding that warrants further study. In general, despite observed difficulty in the fluid cognition domain, all groups performed near the standardization mean in crystallized cognition; furthermore, associations with SDOH for both domains suggested potential modifiable intervention targets. As this study is the first of its size to evaluate cognitive functioning in young adults with PHIV and/or exposed to HIV and ARVs *in utero*, further study is needed to monitor critical, potentially vulnerable aspects of cognitive functioning and identify further sources of resilience in these cohorts as they continue to age.

## Supplementary Information

Below is the link to the electronic supplementary material.

Supplementary material 1 (DOCX 19.5 kb)

Supplementary material 2 (DOCX 22.6 kb)

Supplementary material 3 (DOCX 19.5 kb)

Supplementary material 4 (DOCX 15.7 kb)

Supplementary material 5 (DOCX 22.7 kb)

Supplementary material 6 (DOCX 18.5 kb)

Supplementary material 7 (DOCX 16.5 kb)

Supplementary material 8 (DOCX 17.4 kb)

Supplementary material 9 (DOCX 16.0 kb)

Supplementary material 10 (DOCX 15.3 kb)

Supplementary material 11 (DOCX 19.2 kb)
