# Supplementary material for: Cognitive Functioning and Its Stability Among Young Adults with Perinatal HIV Exposure or Acquisition in a U.S.-Based Longitudinal Cohort Study
Source: AIDS Behav. 2026 Jun 2;30(11):3505–18. doi: 10.1007/s10461-026-05133-2 (PMC13232688; doi:10.1007/s10461-026-05133-2)
Supplement: Supplementary file 1 [file 10461_2026_5133_MOESM10_ESM.docx]

**Supplemental Table 10. Changes in HIV disease severity from entry to Year-3 by HIV status, among YAPHIV with NIH Toolbox assessment at both entry and Year 3**

|  | | | **HIV Status** | |
| --- | --- | --- | --- | --- |
| **Characteristic** | | **Total (N=347)** | **YAPHIV-C**  **(N=207)** | **YAPHIV-non-C (N=140)** |
| Change in CD4 count (cells/mm³) from entry to Year-3 | |  |  |  |
|  | N | 315 | 188 | 127 |
|  | Mean (s.d.) | 31.0 (296.6) | 38.0 (307.2) | 20.7 (281.0) |
|  | Median (Q1, Q3) | 8 (-113, 165) | 14.0 (-111, 159) | 5 (-140, 174) |
| Change in viral load from entry to Year-3 | |  |  |  |
|  | Remained unsuppressed | 77 (22%) | 53 (26%) | 24 (17%) |
|  | Became unsuppressed | 35 (10%) | 20 (10%) | 15 (11%) |
|  | Became suppressed | 48 (14%) | 34 (16%) | 14 (10%) |
|  | Remained suppressed | 156 (45%) | 83 (40%) | 73 (52%) |
|  | Missing | 31 (9%) | 17 (8%) | 14 (10%) |
