# Supplementary material for: Cognitive Functioning and Its Stability Among Young Adults with Perinatal HIV Exposure or Acquisition in a U.S.-Based Longitudinal Cohort Study
Source: AIDS Behav. 2026 Jun 2;30(11):3505–18. doi: 10.1007/s10461-026-05133-2 (PMC13232688; doi:10.1007/s10461-026-05133-2)
Supplement: Supplementary file 2 [file 10461_2026_5133_MOESM11_ESM.docx]

**Supplemental Table 11. Age-corrected cognition composite scores at entry by Social Determinants of Health (SDoH)**

|  | | **Fluid Mean (95% CI)** | **Crystallized Mean (95% CI)** | **Total Mean (95% CI)** |
| --- | --- | --- | --- | --- |
| Race/Ethnicity | Hispanic | 82.6 (80.5, 84.6) | 97.0 (94.8, 99.2) | 87.4 (85.3, 89.5) |
|  | Black Non-Hispanic | 76.9 (75.3, 78.5) | 93.6 (91.9, 95.2) | 82.5 (80.9, 84.1) |
|  | White/Other/Multi-racial Non-Hispanic | 89.8 (85.7, 93.9) | 99.5 (95.8, 103.3) | 93.8 (89.8, 97.7) |
| Highest degree or diploma completed | < high school | 76.6 (74.4, 78.9) | 89.5 (87.2, 91.7) | 79.9 (77.6, 82.1) |
|  | ≥ High school | 81.5 (80.0, 83.0) | 97.8 (96.3, 99.3) | 87.6 (86.1, 89.1) |
| Work for pay | Does not work | 78.5 (76.8, 80.1) | 94.4 (92.6, 96.1) | 83.7 (82.0, 85.4) |
|  | Works part time | 82.3 (79.7, 84.8) | 96.2 (93.7, 98.7) | 87.2 (84.6, 89.7) |
|  | Works full time | 83.0 (80.0, 86.0) | 96.6 (93.8, 99.5) | 88.1 (85.2, 91.0) |
| Annual household/personal income | ≤ 10K | 81.6 (79.5, 83.7) | 96.3 (94.2, 98.4) | 86.9 (84.8, 89.0) |
|  | > 10 - 20K | 81.5 (77.9, 85.2) | 94.9 (91.7, 98.2) | 86.2 (82.8, 89.6) |
|  | > 20K | 85.6 (82.4, 88.8) | 100.3 (96.8, 103.7) | 91.6 (88.2, 94.9) |
|  | Do not know/Rather not answer | 75.4 (73.5, 77.3) | 92.4 (90.4, 94.4) | 80.5 (78.6, 82.5) |
| Type of medical insurance | Public only | 79.2 (77.7, 80.7) | 95.0 (93.4, 96.5) | 84.6 (83.1, 86.1) |
|  | Private only | 86.0 (80.1, 91.9) | 103.5 (98.0, 109.1) | 94.2 (88.8, 99.6) |
|  | Private+ | 81.7 (77.1, 86.3) | 93.4 (88.5, 98.3) | 85.4 (80.5, 90.4) |
|  | Uninsured | 73.5 (64.4, 82.6) | 98.4 (91.1, 105.6) | 82.6 (73.1, 92.1) |
|  | Unaware | 81.8 (78.4, 85.3) | 94.9 (91.5, 98.3) | 86.0 (82.5, 89.4) |
| Any negative financial/legal event | Yes | 79.7 (77.4, 82.1) | 95.6 (93.4, 97.8) | 85.3 (83.0, 87.6) |
|  | No | 80.5 (79.0, 82.0) | 95.6 (94.1, 97.1) | 85.7 (84.2, 87.2) |
| Any negative residential event | Yes | 78.7 (76.4, 81.0) | 94.6 (92.2, 97.0) | 83.8 (81.4, 86.2) |
|  | No | 81.0 (79.5, 82.5) | 96.1 (94.6, 97.6) | 86.4 (84.9, 87.8) |
| Any negative health/death event | Yes | 80.5 (78.9, 82.1) | 95.5 (93.9, 97.2) | 85.6 (84.0, 87.2) |
|  | No | 80.0 (77.9, 82.0) | 95.8 (93.8, 97.7) | 85.6 (83.6, 87.6) |
| Any negative relationship change event | Yes | 79.4 (77.1, 81.8) | 95.9 (93.5, 98.2) | 85.3 (83.0, 87.6) |
|  | No | 80.8 (79.3, 82.3) | 95.6 (94.1, 97.1) | 85.9 (84.4, 87.4) |
| Any negative violence exposure event | Yes | 80.7 (78.1, 83.3) | 97.5 (94.7, 100.2) | 87.2 (84.5, 89.8) |
|  | No | 80.3 (78.8, 81.7) | 95.1 (93.7, 96.6) | 85.2 (83.8, 86.7) |
| Any negative education event | Yes | 77.8 (75.2, 80.4) | 94.4 (92.0, 96.9) | 83.6 (81.1, 86.0) |
|  | No | 81.1 (79.6, 82.5) | 96.0 (94.6, 97.5) | 86.2 (84.8, 87.7) |
| Pearson Correlation Coefficients with Life Events and Support T-scores | |  |  |  |
|  | Total negative events endorsed as YES | -0.074 (-0.148, 0.000) | -0.007 (-0.081, 0.066) | -0.045 (-0.120, 0.030) |
|  | Total life events endorsed as YES | -0.023 (-0.097, 0.052) | 0.041 (-0.033, 0.114) | 0.010 (-0.065, 0.085) |
|  | Emotional support T-score at entry | 0.135 (0.061, 0.206) | 0.091 (0.018, 0.162) | 0.131 (0.057, 0.203) |
|  | Friendship T-score at entry | 0.120 (0.046, 0.192) | 0.088 (0.015, 0.160) | 0.124 (0.050, 0.196) |
|  | Instrumental support T-score at entry | 0.065 (-0.008, 0.139) | -0.009 (-0.082, 0.064) | 0.031 (-0.043, 0.105) |
|  | Self-efficacy T-score at entry | 0.225 (0.154, 0.294) | 0.166 (0.094, 0.236) | 0.232 (0.160, 0.301) |

*Presented above are the mean (95% CI) age-corrected composite score in each category for categorical variables, and the Pearson correlations (95% CI, based on Fisher’s Z transformation) for continuous variables.*
