# Supplementary material for: Cognitive Functioning and Its Stability Among Young Adults with Perinatal HIV Exposure or Acquisition in a U.S.-Based Longitudinal Cohort Study
Source: AIDS Behav. 2026 Jun 2;30(11):3505–18. doi: 10.1007/s10461-026-05133-2 (PMC13232688; doi:10.1007/s10461-026-05133-2)
Supplement: Supplementary file 3 [file 10461_2026_5133_MOESM1_ESM.docx]

**Supplemental Table 1. Unadjusted and adjusted associations of HIV status with age-corrected NIH Toolbox subtest scores at entry**

|  | | **Unadjusted** | | | **Adjusted^a^** | |  |
| --- | --- | --- | --- | --- | --- | --- | --- |
| **Timing and NIH Toolbox Subtest score** | | **Mean (95% CI)** | **Difference vs YAPHEU**  **Beta (95% CI)** | **p-value** | **Difference vs YAPHEU**  **Beta (95% CI)** | **p-value** |  |
| *Fluid Subtest Scores* | | | | | | |  |
|  | **Dimensional Change Card** |  |  |  |  |  |  |
|  | YAPHIV-C | 84.0 (82.1, 85.9) | 3.5 (-0.2, 7.1) | 0.06 | 1.8 (-2.2, 5.9) | 0.37 |  |
|  | YAPHIV-non-C | 86.3 (84.1, 88.5) | 5.8 (2.0, 9.6) | 0.003 | 5.0 (1.1, 8.9) | 0.01 |  |
|  | YAPHEU | 80.5 (77.4, 83.7) | Ref. |  | Ref. |  |  |
|  | **Flanker** |  |  |  |  |  |  |
|  | YAPHIV-C | 77.2 (75.4, 79.1) | -1.7 (-5.3, 1.8) | 0.33 | -1.1 (-5.0, 2.7) | 0.56 |  |
|  | YAPHIV-non-C | 81.8 (79.7, 83.9) | 2.8 (-0.8, 6.5) | 0.13 | 3.5 (-0.3, 7.3) | 0.07 |  |
|  | YAPHEU | 79.0 (76.0, 82.0) | Ref. |  | Ref. |  |  |
|  | **Picture Sequence Memory** | | | | | |  |
|  | YAPHIV-C | 90.6 (89.1, 92.1) | 1.6 (-1.4, 4.5) | 0.30 | 0.6 (-2.6, 3.8) | 0.72 |  |
|  | YAPHIV-non-C | 89.5 (87.8, 91.3) | 0.5 (-2.5, 3.6) | 0.73 | 0.3 (-2.8, 3.5) | 0.83 |  |
|  | YAPHEU | 89.0 (86.5, 91.5) | Ref. |  | Ref. |  |  |
|  | **Pattern Comparison Processing Speed** | |  |  |  |  |  |
|  | YAPHIV-C | 81.2 (79.0, 83.4) | -6.2 (-10.4, -2.0) | 0.004 | -5.9 (-10.4, -1.4) | 0.01 |  |
|  | YAPHIV-non-C | 88.0 (85.5, 90.5) | 0.6 (-3.8, 4.9) | 0.80 | 0.6 (-3.8, 5.0) | 0.79 |  |
|  | YAPHEU | 87.4 (83.9, 91.0) | Ref. |  | Ref. |  |  |
|  | **List Sorting Working Memory** | |  |  |  |  |  |
|  | YAPHIV-C | 92.5 (90.8, 94.2) | -1.5 (-4.8, 1.8) | 0.38 | -1.9 (-5.6, 1.9) | 0.33 |  |
|  | YAPHIV-non-C | 94.4 (92.5, 96.4) | 0.4 (-3.0, 3.9) | 0.81 | 0.5 (-3.2, 4.2) | 0.80 |  |
|  | YAPHEU | 94.0 (91.1, 96.9) | Ref. |  | Ref. |  |  |
| *Crystallized Cognition Subtest Scores* | | | | | | |  |
|  | **Picture Vocabulary** |  |  |  |  |  |  |
|  | YAPHIV-C | 89.5 (88.1, 91.0) | 1.0 (-1.8, 3.8) | 0.47 | 0.8 (-2.2, 3.7) | 0.60 |  |
|  | YAPHIV-non-C | 91.9 (90.2, 93.6) | 3.4 (0.5, 6.3) | 0.02 | 3.6 (0.7, 6.5) | 0.02 |  |
|  | YAPHEU | 88.5 (86.2, 90.9) | Ref. |  | Ref. |  |  |
|  | **Oral Reading Recognition** |  |  |  |  |  |  |
|  | YAPHIV-C | 100.6 (98.2, 103.0) | -2.0 (-6.6, 2.6) | 0.40 | -4.1 (-9.1, 0.9) | 0.11 |  |
|  | YAPHIV-non-C | 100.8 (98.0, 103.6) | -1.8 (-6.6, 3.0) | 0.47 | -2.8 (-7.8, 2.1) | 0.27 |  |
|  | YAPHEU | 102.6 (98.7, 106.5) | Ref. |  | Ref. |  |  |

*YAPHIV-C = YAPHIV with severe disease progression; YAPHIV-non-C =YAPHIV without severe disease progression*

*^a^Adjusted for age at entry, sex, race/ethnicity, and education.*
