# Supplementary material for: Cognitive Functioning and Its Stability Among Young Adults with Perinatal HIV Exposure or Acquisition in a U.S.-Based Longitudinal Cohort Study
Source: AIDS Behav. 2026 Jun 2;30(11):3505–18. doi: 10.1007/s10461-026-05133-2 (PMC13232688; doi:10.1007/s10461-026-05133-2)
Supplement: Supplementary file 4 [file 10461_2026_5133_MOESM2_ESM.docx]

**Supplemental Table 2: Comparing baseline characteristics between participants with vs without year 3 Toolbox data**

|  | | | **Missing Year 3 Data** | |  | |
| --- | --- | --- | --- | --- | --- | --- |
| **Characteristics** |  | **Total (n=739)** | **Yes (n=316)** | **No (n=423)** | **Test Statistics*** | **P_ value** |
| Age (years) at entry NIHT assessment, mean (SD) | | 21.6 (4.1) | 22.0 (4.2) | 21.4 (3.9) | 2.03 | 0.04 |
| Sex at birth | Male | 298 (40%) | 134 (42%) | 164 (39%) | 0.99 | 0.32 |
|  | Female | 441 (60%) | 182 (58%) | 259 (61%) |  |  |
| Race | Black | 487 (66%) | 202 (64%) | 285 (67%) | 1.45 | 0.23 |
|  | White/Other/Multi-racial | 218 (29%) | 101 (32%) | 117 (28%) |  |  |
|  | Missing | 34 (5%) | 13 (4%) | 21 (5%) |  |  |
| Hispanic or Latino | Yes | 221 (30%) | 100 (32%) | 121 (29%) | 1.00 | 0.32 |
|  | No | 509 (69%) | 210 (66%) | 299 (71%) |  |  |
|  | Missing | 9 (1%) | 6 (2%) | 3 (1%) |  |  |
| Highest degree < high school | | 215 (29%) | 79 (25%) | 136 (32%) | 4.94 | 0.08 |
|  | Rather not answer/ missing | 16 (2%) | 8 (3%) | 8 (2%) |  |  |
| Marital status | Never married | 630 (85%) | 270 (85%) | 360 (85%) | 2.82 | 0.42 |
|  | Married | 39 (5%) | 19 (6%) | 20 (5%) |  |  |
|  | Separated/divorced/ widowed | 17 (2%) | 7 (2%) | 10 (2%) |  |  |
|  | Rather not answer | 47 (6%) | 15 (5%) | 32 (8%) |  |  |
|  | Missing | 6 (1%) | 5 (2%) | 1 (0%) |  |  |
| Annual household/personal income | ≤ 10K | 257 (35%) | 103 (33%) | 154 (36%) | 18.41 | < 0.001 |
|  | > 10 - 20K | 98 (13%) | 50 (16%) | 48 (11%) |  |  |
|  | > 20K | 117 (16%) | 66 (21%) | 51 (12%) |  |  |
|  | Do not know or Rather not answer | 261 (35%) | 92 (29%) | 169 (40%) |  |  |
|  | Missing | 6 (1%) | 5 (2%) | 1 (0%) |  |  |
| Type of medical insurance | Public only | 499 (68%) | 206 (65%) | 293 (69%) | 7.08 | 0.21 |
|  | Private only | 33 (4%) | 14 (4%) | 19 (4%) |  |  |
|  | Private+ | 60 (8%) | 32 (10%) | 28 (7%) |  |  |
|  | Uninsured | 14 (2%) | 9 (3%) | 5 (1%) |  |  |
|  | Unaware | 100 (14%) | 38 (12%) | 62 (15%) |  |  |
|  | Rather not answer | 17 (2%) | 6 (2%) | 11 (3%) |  |  |
|  | Missing | 16 (2%) | 11 (3%) | 5 (1%) |  |  |
| Experienced homeless in past 6 months | | 51 (7%) | 25 (8%) | 26 (6%) | 1.83 | 0.40 |
|  | Rather not answer/ missing | 33 (5%) | 14 (5%) | 19 (4%) |  |  |
| Total life events endorsed | N | 722 | 304 | 418 | 0.92 | 0.36 |
|  | Median (Q1, Q3) | 3 (2, 6) | 3.0 (2.0, 5.5) | 3 (1, 6) |  |  |
| Total negative events endorsed | N | 722 | 304 | 418 | 0.75 | 0.45 |
|  | Median (Q1, Q3) | 2 (1, 4) | 2 (1, 4) | 2 (1, 4) |  |  |
| Any negative financial/legal event | | 208 (28%) | 92 (29%) | 116 (27%) | 0.51 | 0.47 |
|  | Missing | 18 (2%) | 12 (4%) | 6 (1%) |  |  |
| Any negative residential event | | 204 (28%) | 87 (28%) | 117 (28%) | 0.08 | 0.78 |
|  | Missing | 24 (3%) | 17 (5%) | 7 (2%) |  |  |
| Any negative health/death event | | 429 (58%) | 190 (60%) | 239 (57%) | 2.23 | 0.14 |
|  | Missing | 18 (2%) | 13 (4%) | 5 (1%) |  |  |
| Any negative relationship change event | | 192 (26%) | 72 (23%) | 120 (28%) | 2.46 | 0.12 |
|  | Missing | 27 (4%) | 15 (5%) | 12 (3%) |  |  |
| Any negative violence exposure event | | 149 (20%) | 65 (21%) | 84 (20%) | 0.16 | 0.69 |
|  | Missing | 21 (3%) | 13 (4%) | 8 (2%) |  |  |
| Any negative education event | | 165 (22%) | 66 (21%) | 99 (23%) | 0.45 | 0.50 |
|  | Missing | 22 (3%) | 13 (4%) | 9 (2%) |  |  |
| Emotional support T-score at entry | N | 734 | 313 | 421 | 0.09 | 0.93 |
|  | Mean (SD) | 44.5 (12.1) | 44.6 (12.4) | 44.5 (11.8) |  |  |
| Instrumental support T-score at entry | N | 733 | 313 | 420 | -1.89 | 0.06 |
|  | Mean (SD) | 45.8 (10.0) | 45.0 (10.2) | 46.4 (9.8) |  |  |
| Friendship T-score at entry | N | 734 | 314 | 420 | -0.63 | 0.53 |
|  | Mean (SD) | 46.1 (12.2) | 45.8 (12.5) | 46.4 (11.9) |  |  |
| Self-efficacy T-score at entry | N | 733 | 313 | 420 | 1.97 | 0.05 |
|  | Mean (SD) | 45.4 (11.0) | 46.3 (10.6) | 44.8 (11.2) |  |  |

** Asymptotic approximations Z score from Wilcoxon rank-sum test for continuous variables; Chi-Square for categorical variables.*
