# Supplementary material for: Cognitive Functioning and Its Stability Among Young Adults with Perinatal HIV Exposure or Acquisition in a U.S.-Based Longitudinal Cohort Study
Source: AIDS Behav. 2026 Jun 2;30(11):3505–18. doi: 10.1007/s10461-026-05133-2 (PMC13232688; doi:10.1007/s10461-026-05133-2)
Supplement: Supplementary file 5 [file 10461_2026_5133_MOESM3_ESM.docx]

**Supplemental Table 3. Unadjusted and Adjusted Associations of HIV status with Changes in Age-corrected NIH Toolbox Subtest Scores from Entry to Year 3**

|  | | **Unadjusted** | | | **Adjusted^a^** | |  |
| --- | --- | --- | --- | --- | --- | --- | --- |
| **Timing and NIH Toolbox Subtest score** | | **Mean (95% CI)** | **Difference vs YAPHEU**  **Beta (95% CI)** | **p-value** | **Difference vs YAPHEU**  **Beta (95% CI)** | **p-value** |  |
| *Fluid Subtest Scores* | | | | | | |  |
|  | **Dimensional Change Card** | | | | | |  |
|  | YAPHIV-C | 3.1 (0.7, 5.6) | -4.0 (-8.8, 0.8) | 0.10 | -4.0 (-9.3, 1.3) | 0.14 |  |
|  | YAPHIV-non-C | 4.9 (1.9, 7.9) | -2.2 (-7.3, 2.8) | 0.39 | -2.4 (-7.7, 3.0) | 0.39 |  |
|  | YAPHEU | 7.1 (3.1, 11.2) | Ref. |  | Ref. |  |  |
|  | **Flanker** |  |  |  |  |  |  |
|  | YAPHIV-C | 2.4 (0.0, 4.9) | -2.0 (-6.7, 2.7) | 0.40 | -4.2 (-9.4, 0.9) | 0.11 |  |
|  | YAPHIV-non-C | -0.8 (-3.8, 2.1) | -5.3 (-10.3, -0.3) | 0.04 | -7.2 (-12.4, -2.0) | 0.01 |  |
|  | YAPHEU | 4.5 (0.5, 8.5) | Ref. |  | Ref. |  |  |
|  | **Picture Sequence Memory** | | | | | |  |
|  | YAPHIV-C | 4.4 (2.5, 6.2) | -0.9 (-4.6, 2.9) | 0.64 | -0.8 (-4.9, 3.4) | 0.71 |  |
|  | YAPHIV-non-C | 7.5 (5.3, 9.8) | 2.3 (-1.7, 6.2) | 0.26 | 2.1 (-2.1, 6.2) | 0.32 |  |
|  | YAPHEU | 5.2 (2.0, 8.5) | Ref. |  | Ref. |  |  |
|  | **Pattern Comparison Processing Speed** | |  |  |  |  |  |
|  | YAPHIV-C | 7.4 (4.7, 10.2) | -3.6 (-8.9, 1.7) | 0.18 | -4.9 (-10.7, 0.9) | 0.10 |  |
|  | YAPHIV-non-C | 3.8 (0.5, 7.2) | -7.2 (-12.8, -1.6) | 0.01 | -8.2 (-14.1, -2.4) | 0.01 |  |
|  | YAPHEU | 11.0 (6.5, 15.5) | Ref. |  | Ref. |  |  |
|  | **List Sorting Working Memory** | |  |  |  |  |  |
|  | YAPHIV-C | 0.8 (-1.6, 3.3) | -1.6 (-6.4, 3.3) | 0.52 | -2.9 (-8.2, 2.5) | 0.30 |  |
|  | YAPHIV-non-C | 1.5 (-1.4, 4.5) | -0.8 (-6.0, 4.3) | 0.75 | -1.9 (-7.3, 3.5) | 0.48 |  |
|  | YAPHEU | 2.4 (-1.8, 6.6) | Ref. |  | Ref. |  |  |
| *Crystallized Cognition Subtest Scores* | | | | | | |  |
|  | **Picture Vocabulary** |  |  |  |  |  |  |
|  | YAPHIV-C | -2.3 (-3.9, -0.7) | -1.3 (-4.5, 1.8) | 0.40 | -0.3 (-3.8, 3.1) | 0.84 |  |
|  | YAPHIV-non-C | -1.1 (-3.1, 0.9) | -0.1 (-3.5, 3.2) | 0.94 | 0.3 (-3.1, 3.8) | 0.85 |  |
|  | YAPHEU | -0.9 (-3.7, 1.8) | Ref. |  | Ref. |  |  |
|  | **Oral Reading Recognition** |  |  |  |  |  |  |
|  | YAPHIV-C | -2.0 (-4.5, 0.5) | -1.2 (-6.1, 3.6) | 0.62 | 0.1 (-5.3, 5.5) | 0.97 |  |
|  | YAPHIV-non-C | 3.9 (0.8, 7.0) | 4.6 (-0.5, 9.8) | 0.08 | 5.6 (0.2, 11.0) | 0.04 |  |
|  | YAPHEU | -0.7 (-4.9, 3.4) | Ref. |  | Ref. |  |  |
|  |  |  |  |  |  |  |  |

*YAPHIV-C = YAPHIV with severe disease progression; YAPHIV-non-C =YAPHIV without severe disease progression*

*^a^Adjusted for age at entry, sex, race/ethnicity, and education.*
