# Supplementary material for: Cognitive Functioning and Its Stability Among Young Adults with Perinatal HIV Exposure or Acquisition in a U.S.-Based Longitudinal Cohort Study
Source: AIDS Behav. 2026 Jun 2;30(11):3505–18. doi: 10.1007/s10461-026-05133-2 (PMC13232688; doi:10.1007/s10461-026-05133-2)
Supplement: Supplementary file 6 [file 10461_2026_5133_MOESM4_ESM.docx]

**Supplemental Table 4. Summary of mediating effect of depressive symptoms on associations between HIV status and changes in age-corrected cognition composite scores from entry to Year 3**

| **Effect of HIV Status** | **Fluid Composite**  **Adj Estimate (95% CI)** | **Crystallized Composite**  **Adj Estimate (95% CI)** | **Total Composite**  **Adj Estimate (95% CI)** |
| --- | --- | --- | --- |
| Comparing YAPHIV-C to YAPHEU | | | |
| Total Effect | -4.54 (-9.36, 0.27) | 0.26 (-3.72, 4.23) | -2.31 (-6.62, 2.00) |
| Natural Direct Effect (NDE) | -4.34 (-9.13, 0.46) | 0.33 (-3.64, 4.31) | -2.18 (-6.49, 2.12) |
| Natural Indirect Effect (NIE) | -0.21 (-0.73, 0.32) | -0.08 (-0.34, 0.19) | -0.13 (-0.50, 0.24) |
| Percentage Mediated | 4.53 (-7.56, 16.62) | -29.67 (-500.14, 440.80) | 5.48 (-12.94, 23.90) |
| Comparing YAPHIV-non-C to YAPHEU | | | |
| Total Effect | -5.54 (-10.07, -1.00) | 2.45 (-1.48, 6.39) | -2.48 (-6.56, 1.59) |
| Natural Direct Effect (NDE) | -5.57 (-10.09, -1.04) | 2.47 (-1.45, 6.38) | -2.53 (-6.58, 1.52) |
| Natural Indirect Effect (NIE) | 0.03 (-0.28, 0.35) | -0.01 (-0.41, 0.38) | 0.05 (-0.41, 0.51) |
| Percentage Mediated | -0.55 (-6.29, 5.19) | -0.52 (-16.65, 15.60) | -2.01 (-21.16, 17.14) |

*Estimates adjusted for age at entry, sex, race/ethnicity, and education.*
