# Supplementary material for: Cognitive Functioning and Its Stability Among Young Adults with Perinatal HIV Exposure or Acquisition in a U.S.-Based Longitudinal Cohort Study
Source: AIDS Behav. 2026 Jun 2;30(11):3505–18. doi: 10.1007/s10461-026-05133-2 (PMC13232688; doi:10.1007/s10461-026-05133-2)
Supplement: Supplementary file 7 [file 10461_2026_5133_MOESM5_ESM.docx]

**Supplemental Table 5: Adjusted associations of HIV disease severity with age-corrected Fluid Cognition subtest scores at entry, among YAPHIV**

|  | | **Dimensional Change Card** | |  | **Flanker** | |  | **Picture Sequence Memory** | |  | **Pattern Comparison Process Speed** | |  | **List Sorting Working Memory** | |
| --- | --- | --- | --- | --- | --- | --- | --- | --- | --- | --- | --- | --- | --- | --- | --- |
|  | | **Adjusted Diff.^a^ (95% CI)** | **P-value** |  | **Adjusted Diff.^a^ (95% CI)** | **P-value** |  | **Adjusted Diff.^a^ (95% CI)** | **P-value** |  | **Adjusted Diff.^a^ (95% CI)** | **P-value** |  | **Adjusted Diff.^a^ (95% CI)** | **P-value** |
| Type of ART regimen at entry | |  |  |  |  |  |  |  |  |  |  |  |  |  |  |
|  | INSTI-based | (ref) | --- |  | (ref) | --- |  | (ref) | --- |  | (ref) | --- |  | (ref) | --- |
|  | PI-based | -3.6 (-7.2, -0.1) | 0.05 |  | -1.6 (-5.1, 1.8) | 0.36 |  | -1.4 (-4.2, 1.5) | 0.34 |  | -3.9 (-8.0, 0.1) | 0.06 |  | 3.0 (-0.3, 6.4) | 0.07 |
|  | NNRTI-based | -1.0 (-5.3, 3.4) | 0.67 |  | 2.3 (-1.9, 6.5) | 0.28 |  | -5.4 (-8.9, -2.0) | 0.002 |  | -3.1 (-8.0, 1.8) | 0.22 |  | -1.2 (-5.3, 2.8) | 0.55 |
|  | Other | -4.5 (-10.2, 1.1) | 0.12 |  | 1.5 (-4.0, 6.9) | 0.60 |  | -1.1 (-5.6, 3.4) | 0.62 |  | -2.2 (-8.6, 4.3) | 0.51 |  | 3.9 (-1.3, 9.2) | 0.14 |
| CD4 count at entry | |  |  |  |  |  |  |  |  |  |  |  |  |  |  |
|  | < 200 cells/mm³ | 1.4 (-3.3, 6.0) | 0.57 |  | -2.1 (-6.6, 2.4) | 0.36 |  | 0.9 (-2.7, 4.6) | 0.61 |  | -2.4 (-7.7, 2.9) | 0.37 |  | -1.7 (-6.1, 2.6) | 0.43 |
|  | 200 - < 500 cells/mm³ | -0.3 (-3.8, 3.1) | 0.85 |  | -0.8 (-4.1, 2.5) | 0.63 |  | -2.0 (-4.7, 0.7) | 0.14 |  | -0.9 (-4.8, 3.0) | 0.65 |  | -3.8 (-7.0, -0.6) | 0.02 |
|  | ≥ 500 cells/mm³ | (ref) | --- |  | (ref) | --- |  | (ref) | --- |  | (ref) | --- |  | (ref) | --- |
| Viral load at entry | |  |  |  |  |  |  |  |  |  |  |  |  |  |  |
|  | < 50 copies/mL | (ref) | --- |  | (ref) | --- |  | (ref) | --- |  | (ref) | --- |  | (ref) | --- |
|  | 50 - < 1000 copies/mL | -2.2 (-6.2, 1.9) | 0.30 |  | -2.4 (-6.4, 1.5) | 0.23 |  | 0.3 (-2.9, 3.6) | 0.84 |  | -0.4 (-5.0, 4.2) | 0.87 |  | 0.8 (-3.1, 4.6) | 0.70 |
|  | ≥ 1000 copies/mL | -2.2 (-5.8, 1.4) | 0.24 |  | -0.8 (-4.3, 2.7) | 0.64 |  | 0.9 (-1.9, 3.8) | 0.52 |  | -4.8 (-8.8, -0.7) | 0.02 |  | -0.0 (-3.4, 3.4) | 1.00 |
| CDC class C at entry | | -5.7 (-9.0, -2.4) | < 0.001 |  | -4.5 (-7.7, -1.4) | 0.01 |  | -1.7 (-4.4, 0.9) | 0.19 |  | -5.5 (-9.2, -1.8) | 0.004 |  | -2.5 (-5.6, 0.5) | 0.10 |
| Past encephalopathy diagnosis | | -5.5 (-11.5, 0.4) | 0.07 |  | -5.0 (-10.6, 0.6) | 0.08 |  | -6.1 (-10.7, -1.5) | 0.01 |  | -6.1 (-12.7, 0.5) | 0.07 |  | -2.1 (-7.6, 3.3) | 0.45 |
| Nadir CD4% prior to study entry | |  |  |  |  |  |  |  |  |  |  |  |  |  |  |
|  | ≥ 15% | (ref) | --- |  | (ref) | --- |  | (ref) | --- |  | (ref) | --- |  | (ref) | --- |
|  | < 15% | -3.0 (-6.0, 0.1) | 0.05 |  | -5.2 (-8.1, -2.3) | < 0.001 |  | 0.9 (-1.5, 3.3) | 0.48 |  | -6.4 (-9.8, -3.0) | < 0.001 |  | -2.3 (-5.1, 0.5) | 0.11 |
| Percent viral load > 1000 (copies/mL) over the 3 years prior to entry | | |  |  |  |  |  |  |  |  |  |  |  |  |  |
|  | 0% | (ref) | --- |  | (ref) | --- |  | (ref) | --- |  | (ref) | --- |  | (ref) | --- |
|  | < 50% | -5.2 (-8.7, -1.6) | 0.004 |  | -2.8 (-6.2, 0.6) | 0.11 |  | 2.1 (-0.7, 4.9) | 0.15 |  | -5.1 (-9.1, -1.1) | 0.01 |  | 1.8 (-1.5, 5.2) | 0.28 |
|  | 50 - < 100% | -2.9 (-7.0, 1.1) | 0.16 |  | -2.8 (-6.7, 1.1) | 0.16 |  | 1.5 (-1.7, 4.6) | 0.37 |  | -3.9 (-8.5, 0.7) | 0.10 |  | 3.8 (0.0, 7.7) | 0.05 |
|  | 100% | -3.9 (-10.1, 2.2) | 0.21 |  | -5.4 (-11.4, 0.6) | 0.08 |  | 1.9 (-2.9, 6.7) | 0.44 |  | -7.2 (-14.1, -0.2) | 0.04 |  | -0.1 (-5.9, 5.7) | 0.97 |
| Age at initiating ART, per year increase | | -0.2 (-0.5, 0.1) | 0.13 |  | -0.1 (-0.4, 0.1) | 0.25 |  | 0.1 (-0.1, 0.3) | 0.38 |  | 0.2 (-0.1, 0.4) | 0.29 |  | -0.1 (-0.3, 0.2) | 0.53 |

*^a^ Estimated difference in adjusted mean age-corrected standard scores comparing a specific exposure group vs reference group for categorical variables, and per one unit increase in continuous variables. A separate general linear regression model was fit for each subtest score, adjusting for age at entry, sex, race/ethnicity, and education.*
