# Supplementary material for: Cognitive Functioning and Its Stability Among Young Adults with Perinatal HIV Exposure or Acquisition in a U.S.-Based Longitudinal Cohort Study
Source: AIDS Behav. 2026 Jun 2;30(11):3505–18. doi: 10.1007/s10461-026-05133-2 (PMC13232688; doi:10.1007/s10461-026-05133-2)
Supplement: Supplementary file 8 [file 10461_2026_5133_MOESM6_ESM.docx]

**Supplemental Table 6: Adjusted associations of HIV disease severity with age-corrected Crystallized Cognition subtest scores at entry, among YAPHIV**

|  | | **Picture Vocabulary** | |  | **Oral Reading Recognition** | |
| --- | --- | --- | --- | --- | --- | --- |
|  | | **Adjusted Diff.^a^**  **(95% CI)** | **P-value** |  | **Adjusted Diff.^a^ (95% CI)** | **P-value** |
| Type of ART regimen at entry | PI-based | 2.3 (-0.4, 5.0) | 0.09 |  | 3.6 (-0.8, 7.9) | 0.11 |
|  | NNRTI-based | 2.4 (-0.8, 5.7) | 0.15 |  | -0.6 (-5.8, 4.7) | 0.84 |
|  | Other | 0.0 (-4.2, 4.3) | 0.99 |  | -1.9 (-8.8, 4.9) | 0.58 |
|  | INSTI-based | (ref) | --- |  | (ref) | --- |
| CD4 count at entry | < 200 cells/mm³ | -2.3 (-5.7, 1.2) | 0.21 |  | -5.3 (-11.0, 0.5) | 0.07 |
|  | 200 - < 500 cells/mm³ | -0.9 (-3.5, 1.7) | 0.49 |  | -2.9 (-7.2, 1.3) | 0.17 |
|  | ≥ 500 cells/mm³ | (ref) | --- |  | (ref) | --- |
| Viral load at entry | < 50 copies/mL | (ref) | --- |  | (ref) | --- |
|  | 50 - < 1000 copies/mL | -2.8 (-5.9, 0.3) | 0.07 |  | -3.4 (-8.4, 1.7) | 0.19 |
|  | ≥ 1000 copies/mL | -1.5 (-4.2, 1.2) | 0.28 |  | -1.1 (-5.6, 3.3) | 0.62 |
| CDC class C at entry |  | -3.5 (-6.0, -1.0) | 0.01 |  | -2.1 (-6.1, 2.0) | 0.32 |
| Past encephalopathy diagnosis |  | -4.1 (-8.5, 0.2) | 0.06 |  | -0.3 (-7.5, 6.9) | 0.93 |
| Nadir CD4% prior to study entry | < 15% | -2.8 (-5.1, -0.5) | 0.02 |  | -2.1 (-5.8, 1.6) | 0.26 |
|  | ≥ 15% | (ref) | --- |  | (ref) | --- |
| Percent of viral load measures > 1000 copies/mL over the 3 years prior to entry | 0% | (ref) | --- |  | (ref) | --- |
|  | < 50% | -0.2 (-2.9, 2.5) | 0.87 |  | 3.4 (-1.0, 7.8) | 0.13 |
|  | 50 - < 100% | -1.5 (-4.6, 1.5) | 0.32 |  | 0.8 (-4.2, 5.8) | 0.76 |
|  | 100% | -3.6 (-8.3, 1.0) | 0.13 |  | 0.3 (-7.4, 7.9) | 0.95 |
| Age at initiating the first ART, per year increase |  | 0.0 (-0.1, 0.2) | 0.65 |  | -0.0 (-0.3, 0.3) | 0.82 |

*^a^ Estimated difference in adjusted mean age-corrected standard scores comparing a specific exposure group vs reference group for categorical variables, and per one unit increase in continuous variables. A separate general linear regression model was fit for each subtest score, adjusting for age at entry, sex, race/ethnicity, and education.*
