# Supplementary material for: Cognitive Functioning and Its Stability Among Young Adults with Perinatal HIV Exposure or Acquisition in a U.S.-Based Longitudinal Cohort Study
Source: AIDS Behav. 2026 Jun 2;30(11):3505–18. doi: 10.1007/s10461-026-05133-2 (PMC13232688; doi:10.1007/s10461-026-05133-2)
Supplement: Supplementary file 9 [file 10461_2026_5133_MOESM7_ESM.docx]

**Supplemental Table 7. Adjusted associations of historical HIV disease severity with age-corrected cognition composite scores at entry, among YAPHIV rolled over from AMP**

|  | | **Fluid** | |  | **Crystallized** | |  | **Total** | |
| --- | --- | --- | --- | --- | --- | --- | --- | --- | --- |
|  | | **Adjusted Mean Diff.^a^ (95% CI)** | **P-value** |  | **Adjusted Mean Diff.^a^ (95% CI)** | **P-value** |  | **Adjusted Mean Diff.^a^ (95% CI)** | **P-value** |
| Nadir CD4% prior to study entry | |  |  |  |  |  |  |  |  |
|  | ≥ 15% | (ref) | --- |  | (ref) | --- |  | (ref) | --- |
|  | < 15% | -3.9 (-7.9, 0.1) | 0.06 |  | -2.0 (-6.0, 2.1) | 0.34 |  | -3.5 (-7.5, 0.5) | 0.09 |
| Percent of viral load measures > 1000 copies/mL over the 3 years prior to entry | | | | | | | | | |
|  | 0% | (ref) | --- |  | (ref) | --- |  | (ref) | --- |
|  | < 50% | -1.5 (-6.3, 3.3) | 0.54 |  | 2.7 (-2.1, 7.6) | 0.27 |  | 0.5 (-4.3, 5.3) | 0.83 |
|  | 50 - < 100% | 2.2 (-3.0, 7.4) | 0.41 |  | 2.1 (-3.2, 7.3) | 0.44 |  | 2.1 (-3.2, 7.3) | 0.44 |
|  | 100% | -8.0 (-18.7, 2.8) | 0.15 |  | 0.5 (-10.6, 11.6) | 0.93 |  | -4.7 (-15.4, 6.0) | 0.39 |
| Age at ART initiation, per year increase | | 0.2 (-0.6, 0.9) | 0.65 |  | 0.2 (-0.6, 1.0) | 0.62 |  | 0.2 (-0.5, 1.0) | 0.59 |

*^a^ Estimated difference in adjusted mean age-corrected composite scores, based on a separate general linear regression model for each score, adjusting for age at entry, sex, race/ethnicity, and education.*
