# Supplementary material for: Cognitive Functioning and Its Stability Among Young Adults with Perinatal HIV Exposure or Acquisition in a U.S.-Based Longitudinal Cohort Study
Source: AIDS Behav. 2026 Jun 2;30(11):3505–18. doi: 10.1007/s10461-026-05133-2 (PMC13232688; doi:10.1007/s10461-026-05133-2)
Supplement: Supplementary file 10 [file 10461_2026_5133_MOESM8_ESM.docx]

**Supplemental Table 8: Adjusted associations of historical HIV disease severity with age-corrected Fluid Cognition subtest scores at entry, among YAPHIV rolled over from AMP**

|  | | **Dimensional Change Card** | |  | **Flanker** | |  | **Picture Sequence Memory** | | **Pattern Comparison Process Speed** | | **List Sorting Working Memory** | |
| --- | --- | --- | --- | --- | --- | --- | --- | --- | --- | --- | --- | --- | --- |
|  | | **Adj. Mean Diff.^a^ (95% CI)** | **P- value** |  | **Adj. Mean Diff.^a^ (95% CI)** | **P- value** |  | **Adj. Mean Diff.^a^ (95% CI)** | **P- value** | **Adj. Mean Diff.^a^ (95% CI)** | **P- value** | **Adj. Mean Diff.^a^ (95% CI)** | **P- value** |
| Nadir CD4% prior to study entry | |  |  |  |  |  |  |  |  |  |  |  |  |
|  | ≥ 15% | (ref) | --- |  | (ref) | --- |  | (ref) | --- | (ref) | --- | (ref) | --- |
|  | < 15% | -2.4 (-6.9, 2.2) | 0.31 |  | -5.9 (-10.3, -1.6) | 0.01 |  | 1.4 (-1.9, 4.7) | 0.40 | -4.8 (-9.7, 0.2) | 0.06 | -1.9 (-6.2, 2.3) | 0.38 |
| Percent of viral load measures > 1000 copies/mL over the 3 years prior to entry | | | | | | | | | |  |  |  |  |
|  | 0% | (ref) | --- |  | (ref) | --- |  | (ref) | --- | (ref) | --- | (ref) | --- |
|  | < 50% | -3.2 (-8.6, 2.2) | 0.24 |  | -1.7 (-7.0, 3.6) | 0.53 |  | 2.6 (-1.2, 6.4) | 0.18 | -3.5 (-9.4, 2.4) | 0.24 | 2.0 (-3.1, 7.1) | 0.45 |
|  | 50 - < 100% | -0.5 (-6.3, 5.3) | 0.87 |  | -1.1 (-6.8, 4.6) | 0.69 |  | 5.6 (1.6, 9.7) | 0.01 | -1.3 (-7.7, 5.0) | 0.68 | 5.5 (0.0, 11.0) | 0.05 |
|  | 100% | -2.6 (-14.9, 9.7) | 0.68 |  | -9.3 (-21.4, 2.9) | 0.13 |  | 0.9 (-7.7, 9.5) | 0.84 | -9.4 (-22.8, 4.1) | 0.17 | 1.7 (-10.0, 13.4) | 0.78 |
| Age at ART initiation, per year increase | | 0.4 (-0.4, 1.3) | 0.33 |  | -0.2 (-1.0, 0.6) | 0.65 |  | 0.2 (-0.5, 0.8) | 0.60 | -0.2 (-1.1, 0.7) | 0.66 | 0.9 (0.1, 1.7) | 0.03 |

*^a^ Estimated difference in adjusted mean age-corrected subtest scores, based on a separate general linear regression model for each score, adjusting for age at entry, sex, race/ethnicity, and education.*
