# Supplementary material for: Cognitive Functioning and Its Stability Among Young Adults with Perinatal HIV Exposure or Acquisition in a U.S.-Based Longitudinal Cohort Study
Source: AIDS Behav. 2026 Jun 2;30(11):3505–18. doi: 10.1007/s10461-026-05133-2 (PMC13232688; doi:10.1007/s10461-026-05133-2)
Supplement: Supplementary file 11 [file 10461_2026_5133_MOESM9_ESM.docx]

**Supplemental Table 9: Adjusted associations of historical HIV disease severity with age-corrected Crystallized Cognition subtest scores at entry, among YAPHIV rolled over from AMP**

|  | | **Picture Vocabulary** | |  | **Oral Reading Recognition** | |
| --- | --- | --- | --- | --- | --- | --- |
|  | | **Adjusted Mean Diff.^a^ (95% CI)** | **P-value** |  | **Adjusted Mean Diff.^a^ (95% CI)** | **P-value** |
| Nadir CD4% prior to study entry | |  |  |  |  |  |
|  | ≥ 15% | (ref) | --- |  | (ref) | --- |
|  | < 15% | -1.9 (-5.3, 1.5) | 0.28 |  | -1.7 (-6.9, 3.5) | 0.52 |
| Percent of viral load measures > 1000 copies/mL over the 3 years prior to entry | | | | | | |
|  | 0% | (ref) | --- |  | (ref) | --- |
|  | < 50% | 0.2 (-3.9, 4.4) | 0.91 |  | 5.0 (-1.3, 11.2) | 0.12 |
|  | 50 - < 100% | 0.1 (-4.4, 4.5) | 0.97 |  | 3.6 (-3.1, 10.4) | 0.29 |
|  | 100% | 0.4 (-9.0, 9.8) | 0.93 |  | 0.2 (-14.1, 14.5) | 0.98 |
| Age at ART initiation, per year increase | | 0.2 (-0.5, 0.8) | 0.62 |  | 0.2 (-0.7, 1.2) | 0.62 |

*^a^ Estimated difference in adjusted mean age-corrected subtest scores, based on a separate general linear regression model for each score, adjusting for age at entry, sex, race/ethnicity, and education.*
